# A representative dataset on Czech and Slovak pre-Russia–Ukraine-war attitudes to the West and to the Russian Federation

**DOI:** 10.1016/j.dib.2023.109871

**Published:** 2023-11-29

**Authors:** Jiří Čeněk, Marek Čejka, Vladimir Đorđević, Martin Hrabálek, Petra Mlejnková, Ondřej Mocek

**Affiliations:** a Mendel University in Brno, Faculty of Regional Development and International Studies, Czechia; b Ambis University, Czechia; c Masaryk University, Faculty of Social Studies, Czechia

**Keywords:** Popular attitudes, Czechia, Slovakia, Pro-western, Anti-western, Pro-Russian, Anti-Russian

## Abstract

This article presents a comprehensive dataset and its associated data collection methodology aimed at understanding public opinion and attitudes across diverse socio-political dimensions in Czechia and Slovakia. The dataset covers a wide array of variables, including socio-demographic variables, media consumption patterns, scales measuring attitudes and ideology, evaluations of political statements, and reactions to political billboards. The dataset is structured into folders containing raw data, metadata, research samples, analyses, and visual stimuli used in the study. The raw data files encompass original survey responses received from participants, while the metadata provides essential details about variables, their coding, scaling, and question formulation. The survey contains data from more than 1200 Czech and Slovak participants. The samples are representative by gender, age, education level, region, and place of residence size.

Researchers utilizing this dataset have the opportunity to explore public opinion dynamics, ideological tendencies, and responses to political stimuli. The dataset's extensive and multifaceted nature offers a valuable resource for various analyses, allowing researchers to delve into factors shaping political orientations, public sentiment towards specific political narratives, and the impact of visual stimuli on political perceptions. Moreover, the dataset's inclusion of both Czechia and Slovakia provides a comparative dimension, enabling cross-country analyses and insights into potential socio-political divergences or convergences within the region. Its accessibility and comprehensiveness make it a valuable asset for a wide range of research endeavors across political science, sociology, psychology, and related disciplines.

Specifications Table

**Table:** 

Subject	Psychology
Specific subject area	attitudes to major geo-political entities; ideology scales; political statements
Data format	Raw
Type of data	Table (Excel), Image (.jpg)
Data collection	Data was collected via an online survey in August 2021 by a marketing research agency NMS (https://nms.global/en/). Quota sampling was used with quotas imposed on gender, age group, gender-age combination, education level, region and size of place of residence (see Quotas file in the repository). Apart from items created by the authors, several psychological scales previously developed by other authors were used. More specifically, the scales of social conservatism (three items)[1], authoritarianism measured by child-rearing values (four items)[2], authoritarian aggression and submission subscale of the right-wing authoritarianism scale (seven items)[3], national identity (three items), European identity (three items)[4], nationalism (three items)[5] are included in the dataset.
Data source location	·Institution: Mendel University in Brno ·City/Town/Region: Brno ·Country: Czechia ·Latitude and longitude (and GPS coordinates, if possible) for collected samples/data: 49.217625, 16.614576
Data accessibility	Repository name: OSF Data identification number: 10.17605/OSF.IO/TFC59 Direct URL to data: https://osf.io/tfc59/

## 1 Value of the Data


 

• This dataset might help researchers from the fields of political and cross-cultural psychology, sociology, political science, or international relations to better understand links between sociodemographic variables (gender, age, etc.), psychological constructs commonly used in political psychology such as authoritarianism, social conservatism, European and national identity, nationalism, or attitudes to types of social organization (liberalism, capitalism, communism, democracy, authoritarianism), and ecologically valid statements about the main geopolitical entities in the Czech and Slovak context (NATO, EU, USA, Russian Federation).
• This dataset also includes set of “political billboards” (combination of statements about geopolitical entities, party acronyms and people - avatars of unknown politicians) that were evaluated by the positive/negative feelings they elicit in the participants and by the probability that the participants would vote for party promoting itself by such a billboard.
• The dataset is unique mainly due to the timing of the data collection. The data were collected in the summer of 2021. In other words, they were collected before the Russian invasion of Ukraine and the ensuing shift in both Czech and Slovak attitudes. Therefore, the dataset can serve as basis for future research on potential changes in the attitudes of both national groups.
• Potential replication studies could shed light on the dynamics of the attitudes to the main geopolitical entities in the European context.

## 2 Data Description

The dataset and the metadata is available at OSF repository (https://osf.io/tfc59/). The content is structured into several folders.

### 2.1 Folder Structure

#### 2.1.1 Raw data folder

This folder contains two raw data files *Full_data_Czechia.xlsx* and *Full_data_Slovakia.xlsx.* These two files contain original data as received by the data collecting company. The entire data is in numeric format. The folder also contains a metadata file *Variables_and_scaling.xlsx* with variable names, numeric coding, scaling, and their English translation. In sheets *Variables_Czechia* and *Variables_Slovakia* you can find variable names, question numbers, formulation of questions, measurement level and English translation. Sheets *Scaling_Czechia* and *Scaling_Slovakia* link numeric coding of variables in the raw data files to their wording. English translation of the original Czech and Slovak wording of response options is also included in these sheets. For those, who would like to see the exact appearance of the questionnaire, files *Questionnaire_CZ_screenshots* (and *Questionnaire_SK_screenshots*) contain screenshots of the questionnaire as presented to the participants.

It is important to note that there are variations in certain questions between the samples. Specifically, the variable ‘*VAHY*’ (weight) represents sample weights calculated based on the general population of Czechia and Slovakia, respectively. Additionally, two variables asking respondents about the region they reside in (variable numbers 11 and 12) differ in both samples. Questions related to income are formulated in the respective currencies of each country (Czech Crown in Czechia, Euro in Slovakia; variable numbers 14 and 15). Moreover, there are inquiries about media consumption, covering TV, radio, newspapers, magazines, and online sources. The questions regarding media consumption have variable names starting with ‘*news_source_*’. These questions list prominent media outlets with significant audience reach in each respective country, and the specific media mentioned in the questions are enclosed in square brackets. Finally, one of the billboards (nr. 13) mentions the name of the capital (Prague/Bratislava) of the respective country.

#### 2.1.2 Research sample folder

This folder contains a *Quotas.xlsx* file with quota tables for both samples. Each table includes columns for quotas, collected participants, and weights. For details on the research sample see the next section.

#### 2.1.3 Analyses folder

This folder contains RStudio compatible R scripts and workspaces that were created to make the data more accessible and easier to use for other researchers. All codes should run after opening the files in RStudio and changing the pathway of the input files to actual pathways. The folder contains *data_preprocessing.Rmd* that can be used as a whole, or be adjusted based on the intentions of the researcher. It takes numeric data matrices from both samples as input and performs a series of data preprocessing and data cleaning operations. More specifically, it combines both matrices, sets common factor levels of factor variables, cleans and unifies variables connected with voting behavior, recodes scales that need to be recoded to work correctly (conservatism, authoritarianism), calculates means of some of the psychological scales, performs outlier analyses.

Workspace *workspace_1_reliabilities.RData* and related script *scales_reliabilities*.Rmd performs psychometric analysis of scales with higher number of items. More specifically, it performs exploration of correlations across items, multivariate outlier detection, multigroup invariance testing and calculation of reliabilities (alpha, omega) of Social and economic conservatism [1] and Right-Wing Authoritarianism [3] scales.

Workspace *workspace_2_for_analysis.RData* and related *pca.Rmd* performs a Principal Component analysis (PCA) of Political Statements questions (for more details on the creation process of these statements, their administration and PCA results see the respective subsection of Experimental Design, Materials and Methods section).

#### 2.1.4 Billboards folder

This folder contains 46 pictures - billboards (23 for each country) that were used in the last section of the questionnaire (for more details on the creation process of these pictures, and their administration see the respective subsection of Experimental Design, Materials and Methods section).

### 2.2 Dataset Description

The dataset can be divided into several subsections: socio-demographic variables, media consumption, scales of attitudes and ideology, evaluation of political statements and evaluation of political billboards. To help the researchers potentially using this data to get oriented we mention the column names of the variables in the raw data files (*Full_data_Czechia/Slovakia*). The column names sometimes differ in Czechia and Slovakia due to a different number of media-related questions. The details of the question wording and scaling can be found in *Variables_and_scaling* file.

#### 2.2.1 Socio-demographic variables

We asked a number of variables that are useful for understanding the background of the participants to be able to subsequently link this background to their attitudes. We asked for all the commonly asked questions, namely gender, age, age group, education, region, municipality size, income, perceived social status (Cols. *E-Q*), household size and number of children (Cols. *GT-GU* in Czechia; *FS-F*T in Slovakia). We also asked about their religious affiliation and practices (Cols. *GI-G*S in Czechia; *FH-FR* in Slovakia), and political preferences and voting behavior (Cols. *GW-GZ* in Czechia; *FV-FZ* in Slovakia).

#### 2.2.2 Media consumption

Media people consume can play a role in the formation of their political attitudes. We included a set of binary variables (yes/no) regarding various media sources: TV and radio stations, newspapers and magazines and online news pages. This set of questions differs between both countries - people in Czechia consume different media compared to the Slovaks. The names of media consumption variables start with “*news_source_*”, followed by the media type (“_*TV_*”, “_*radio_*”, “_*paper_*”, “_*magazines_*”, “_*online_*”). Apart from the binary variables, each media type has open questions where respondents wrote media that they follow but were not in the list. One question (*media_intensity*) asks the respondents about how much time they spend using media per day.

#### 2.2.3 Scales of attitudes and ideology

Several scales of attitudes and ideology are part of the dataset. They can play a role of both independent (or as moderators/mediators) and dependent variables in potential analyses. If the researchers using this dataset want to create some aggregated scores of scales with multiple items, it can be, with one exception of scale of authoritarianism measured by child-rearing values (described below), suggested 1) to calculate correlations and test the reliability of subset of questions they want to aggregate, 2) aggregate the data by averaging the item scores. Scale of Social and Economic Conservatism [1] (SECS; in data the variables start with “*secs_*”) measures the level of participants' agreement with topics associated with issues such as abortion rights and gender equality (social conservatism) or progressive taxation and role of the state in economy (economic conservatism). It is a 15-item scale with 0–100 thermometer response format.

Level of liberalism of the respondents can be also estimated by a self-generated standalone 7-point (very conservative - very liberal) question whether the respondents hold conservative or liberal stances (*ideol_LC_self_id*). Several other items that are commonly used in political psychology are included in the dataset. Standalone 5-point self-generated question on right-wing/left-wing preferences (right wing - left wing; *right_left_pref*). Sets of 7-point questions asking respondents to evaluate their national identity (three items; variable names start with “*nation_ident_*”) [4], European identity (three items; “*euro_ident_*”) [4], and five thermometer questions related to attitudes toward communism, capitalism, democracy, liberalism and authoritarianism (“*regim_attit_*”) [6] are included in the dataset.

Two of the scales focus on authoritarianism. More specifically, the dataset includes seven items of Authoritarian Aggression and Submission subscale of the Right-wing Authoritarianism (RWA) scale (coded as “*rwa_*”; 7-point, definitely disagree - definitely agree) with items like “*Our country urgently needs a strong leader who will do everything necessary to eradicate new radical ideologies and changes in our way of life.*” [3] and four items on authoritarianism measured by child-rearing values (in dataset as “*authorit_scale_*”) [2]. The latter scale adopts a different approach to measure authoritarianism than RWA. Instead of asking directly about the level of agreement with authoritarianism-related statements it asks the respondents “*Which of the following traits should children be raised with?*” and presents them with a choice between an “authoritarian” and “non-authoritarian” response such as “*Independence or respect for elders*” or “*Obedience or self-confidence*”. The respondents have five response options. They can choose one (e.g., *independence*), or another (e.g., *respect for elders*), or they can answer “*both*”, “*none*”, “*not sure*”. This scale is constructed as additive. If the researcher wants to work with it in the same fashion as the authors of the original study [2] they should recode the authoritarian responses as “1”, non-authoritarian responses as “0”, and the remaining options as 0.5.

Finally, the level of nationalism can be estimated by three 5-point Likert-type questions such as “*Other countries do things well too, but not as well as the Czech Republic/Slovakia.*” (“*nationalism_*”) [5].

#### 2.2.4 Evaluation of political statements

The dataset contains 25 pro-/anti-Western and pro-/anti-Russian statements such as “Russia doesn't need to wage wars of conquest. It is just protecting its independence and sovereignty.” (pro-Russian), “Russians do not deal with other Slavs as brothers, but dishonestly and selfishly.” (anti-Russian), “Czech/Slovak diplomacy must try together with its European partners to prevent the USA from leaving European space and succumbing to isolation.” (pro-Western), or the “EU is a fascist organization, NATO is a criminal organization.” (anti-Western). The participants evaluated the statements on a 6-point Likert-type scale (definitely disagree - definitely agree) with additional “I don't know, I can't judge” option. The process of creation of the statements is described in the subsection of Experimental Design, Materials and Methods section.

#### 2.2.5 Evaluation of political billboards

Finally, the dataset contains a set of participants' reactions to 23 pictures-billboards. Each billboard was evaluated by two questions. Question on elicitation of positive or negative feelings (*Billboard evokes in me.*; 0 = *very negative feelings*, 10 = *very positive feelings*; coded as *B01-B23A*), and a question on the probability they would vote for a party represented by the billboard (*What is the likelihood that you would vote for this political party based on this billboard?; 0–100 %*; coded as *B01-B23B*). The process of creation of the billboards and their parameters is described in the subsection of Experimental Design, Materials and Methods section.

### 2.3 Research Sample

Quota sampling method was used with quotas imposed on gender, age group, gender-age combination, education level, region and size of place of residence. The size of the quotas were determined using population statistics of the Czech and Slovak Statistical office. For details on quotas, acquired samples and quota-sample differences see excel file *Quota*s in repository. In total, 603 Czech and 602 Slovak respondents were gathered. See Table 1 for sample descriptives.

**Table 1:** Description and frequency distribution of basic socio-demographic variables.

		Czechia		Slovakia	
Variable	Level	*N*	Percent	*N*	Percent
Gender	male	303	50.25	292	48.50
	female	300	49.75	310	51.50
Age	18-24	57	9.45	67	11.13
	25-34	122	20.23	129	21.43
	35-44	145	24.05	152	25.25
	45-54	126	20.90	122	20.27
	55-64	153	25.37	132	21.93
Education	no education	2	0.33	5	0.83
	elementary school	32	5.31	31	5.15
	high school - no diploma	213	35.32	227	37.71
	high school with diploma	232	38.47	235	39.04
	Bachelor	40	6.63	16	2.66
	Master and higher	84	13.93	88	14.62

## 3 Experimental Design, Materials and Methods

### 3.1 Materials

The questionnaire was administered between August 13 and 20 2021 using CAWI method of data gathering by NMS Market Research agency [7].

#### 3.1.1 Socio-demographic, media and attitudes and ideology questions

The socio-demographic and media questions were derived from the standard pool of questions used by the agency in Czechia and Slovakia.

The attitudes and ideology scales were translated to both languages from English original [1], [2], [3], [4], [5], [6] using a backtranslation method with two independent translators and an editor for each language. The translation was conducted as part of a previous large-scale Sinophone survey [8] that gathered representative data in 13 European countries.

It should be noted that the SECS was adapted to a larger extent than just a literal translation. The original scale [1] was developed for US samples and some of the questions do not reflect the nature of conservatism-liberalism as understood by Europeans (most prominently “*carrying a gun*”, because in the majority of European countries gun ownership by the general public is very limited and heavily regulated). The following items were retained from the original scale with no changes [1]: *Welfare benefits, Religion, Patriotism, Traditional marriag*e, *Traditional values*. Some items were adapted to be more understandable for European participants. More specifically, *Abortion* was replaced with *Abortion rights, Immigration* changed to *Migration*. The rest of the items were discarded and some or replaced with conceptually similar items. Some items, for example *Gender equality*, or *Renewable sources of energy* were newly added. In Table 2 we listed all items administered as SECS scale. In the last column “S” and “E” addendums indicate whether an item is part of Social or Economic Subscale, the “*” indicates a reverse scored item (the higher the score, the lower conservatism). Please note that in the raw data files, the reverse scored items are not reversed. The item reversion is conducted in *data_preprocessing.Rmd*, lines 331–341. The “†” indicates items that are invariant across both samples (see section Invariance and reliability).

**Table 2:** Adapted SECS scale.

Item label (raw data)	Item wording	Subscale, reversion
secs_scale_1	Abortion rights	S*
secs_scale_2	Religion	S
secs_scale_3	Welfare benefits	E*
secs_scale_4	Traditional marriage	S†
secs_scale_5	Traditional values	S†
secs_scale_6	Single-parent family	S*
secs_scale_7	Patriotism	S†
secs_scale_8	Same-sex marriage	S*
secs_scale_9	Strong role of government in economy	E*†
secs_scale_10	Migration	S*
secs_scale_11	Gender equality	S*
secs_scale_12	Compulsory vaccination	S*
secs_scale_13	Higher tax rates for people with higher income	E*†
secs_scale_13s	Renewable sources of energy	E*†
secs_scale_14	Nuclear power	E

#### 3.1.2 Political statements

We gathered a collection of political statements that appeared in the public space of both countries between 2020 and 2021. We have chosen this approach over statements created in an ad-hoc manner due to higher ecological validity of the research. We searched through social network profiles, press announcements, and webpages of political parties, political movements, and individual politicians from all segments of the political spectrum with focus on both positive and negative statements related to Russia, EU, and NATO.

In the second stage, we asked eight experts from both countries to conduct an initial qualitative classification of each statement (categories: *Nationalist, Populist, Slavic unity advocate, Pro-Russian, Anti-Russian, Anti-Slavic, Anti-European, Eurosceptic, Pro-European, Eurooptimistic, Pro-American, Anti-American, Pro-alliance (NATO), Anti-alliance (NATO)*) and discarded all statements that were ambiguous, out of the scope of the research, or those too complex for respondents to sensibly evaluate on a response scale.

We ended up with a set of 25 statements such as “Russia doesn't need to wage wars of conquest. It is just protecting its independence and sovereignty.” (pro-Russian), “Russians do not deal with other Slavs as brothers, but dishonestly and selfishly.” (anti-Russian), “Czech/Slovak diplomacy must try together with its European partners to prevent the USA from leaving European space and succumbing to isolation.” (pro-Western), or the “EU is a fascist organization, NATO is a criminal organization.” (anti-Western). It must be noted that some of these statements can have multiple semantic components. For example, “We need to exit NATO - It drags us into provocations against Russia.” Has a clearly both anti-Western and pro-Russian meaning, and “Neither East, nor West, but Czechia/Slovakia!” has anti-Western, anti-Eastern, as well as nationalist components.

The response scale of these statements is a six-point Likert scale (1 = Strongly disagree, 6 = Strongly agree, including an “I don't know” option). The full list of the statements can be found in *Variables_and_scaling* file.

#### 3.1.3 Political billboards

**Fig. 1:**
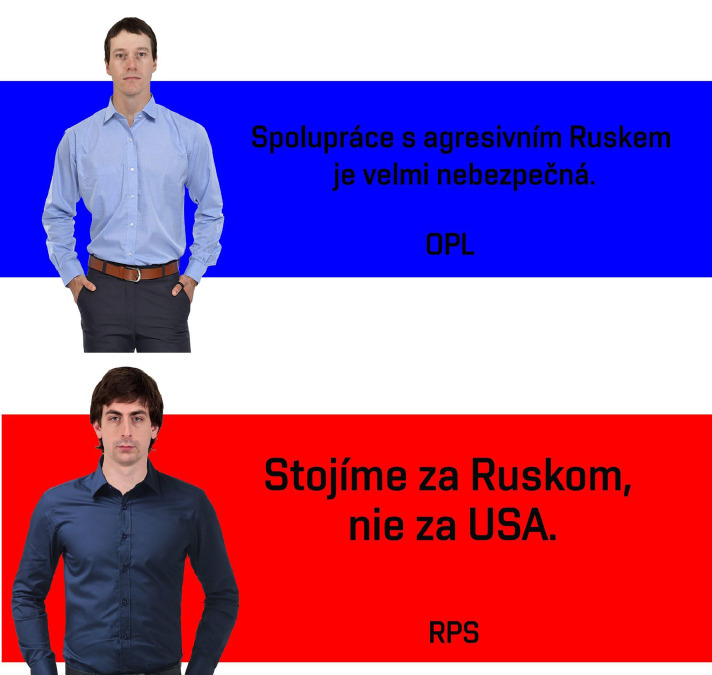
Billboards. Examples of Czech anti-Russian [*Cooperation with aggressive Russia is very dangerous*] and Slovak pro-Russian [*We stand with Russia, not with the USA*] Billboard.

To be able to explore how the respondents perceive more complex, graphical and ecologically valid stimuli, we created a set of 23 “billboards”, similar to the billboards people can see in Czech and Slovak streets during the election campaigns. The billboards were composed of several elements (see Fig. 1) - face, body, statement, “party abbreviation”, and color.

(a) **Face**: The faces were taken from Chicago Face database [9]. To reduce potential effects of ethnicity, gender, age and specific face characteristics we filtered the available faces according to the following criteria: we have chosen only faces of caucasian males (male and white probabilities > 0.90) between 28 and 45 years of age, attractivity rating between 3.92 and 2.09 (1.5 SD from the mean) and unusualness rating between 3.27 and 2.09 (again, 1.5 SD from the mean).
(b) **Body**: Photos of bodies were taken from free web databases (CC-BY-NC or lower) with limitations to business casual/smart casual dress code.
(c) **Statement**: Part of the political statements we gathered (see the previous section for details) had a character suitable for using in billboards (relatively short, 1–2 sentences). E. g. the statements on the billboards in Fig. 1 say “*Cooperation with aggressive Russia is very dangerous.*” (anti-Russian) and “*We stand with Russia, not with the USA.*” (pro-Russian, anti-western). English translation of all statements is in the *Variables_and_scaling* file.
(d) **Party abbreviation**: To make the billboards more “believable” we added abbreviations of parties under the statements. They are composed of three (e.g. “*EDS”*), or four (“*SSDP”*) letters, or combination of letters and numbers (“*HR100*”). This approach was chosen because abbreviations of most real-world political parties in both countries have a similar format [10].
(e) **Color**: To make the billboards more visually appealing, each billboard has a background color (red, green, blue, orange, lila, grey, or brown) that is placed on left, right, or the center of the billboard space.

All elements of the billboards were combined using Adobe Photoshop CC 2019 [11].

### 3.2 Analyses

We conducted several analyses to explore the data. More specifically, we conducted an invariance testing and reliability estimation of scales with higher number of items (SECS, RWA; file *scales_reliabilities.Rmd*) and Principal Components Analysis (PCA) of evaluation of political statements and billboards (file *pca.Rmd*).

#### 3.2.1 Invariance and reliability

For testing whether SECS and RWA scales work well in both countries, we performed invariance testing using the MGCFA approach [12]. In the case of SECS, we first tested a two-factor model with all items. This model showed insufficient model fit. Based on the item loadings, we reduced the number of items to three items (nr. 4, 5, 7) in social and three items (9, 13, 14) in economic factor. This configural model was subsequently tested and showed good [13] model fit (RMSEA = 0.064, CFI = 0.972, TLI = 0.948, SRMR = 0.032). Moreover, both metric (RMSEA = 0.057, CFI = 0.972, TLI = 0.958, SRMR = 0.034) and scalar (RMSEA = 0.067, CFI = 0.954, TLI = 0.942, SRMR = 0.042) invariance was established across the samples. In the next step, we tested for reliability of both subscales. The social conservatism subscale shows good reliability in both Czechia (alpha = 0.773, omega = 0.775) and Slovakia (alpha = 0.751, omega  = 0.772). The economic conservatism subscale shows poor reliability in Czechia (alpha = 0.383, omega = 0.380) and Slovakia (alpha = 0.562, omega = 0.573).

Furthermore, we applied the same procedure to the RWA scale. The configural model fits well to the data (RMSEA = 0.092, CFI = 0.949, TLI = 0.923, SRMR = 0.038). Both metric (RMSEA = 0.085, CFI = 0.947, TLI = 0.935, SRMR = 0.044) and scalar (RMSEA = 0.067, CFI = 0.937, TLI = 0.934, SRMR = 0.048) invariance was established across the samples. The scale shows very good reliability in both Czechia (alpha = 0.866, omega = 0.867) and Slovakia (alpha = 0.814, omega = 0.814).

The MGCFA approach is not suitable for scales with lower number of items. In the case of these scales we simply calculated the alpha reliability. National identity shows very good reliability in both Czech (alpha = 0.896) and Slovak (alpha = 0.889) samples. This is also the case of European identity with very good reliability in the Czech (alpha = 0.880) and Slovak (alpha = 0.899) sample. Finally, regarding nationalism, the reliability can be considered good in both Czech (alpha = 0.757) and Slovak (alpha = 0.705) samples.

#### 3.2.2 PCA

We conducted PCA on political statements. The analysis revealed that the statements form two components: 1) anti-western and pro-Russian (items n1–n16) and 2) pro-western and anti-Russian (items p17–p31). The item loadings are relatively high with minimal cross-loadings.

Results of PCA of political billboards show more complicated patterns with possible 2 or 3 principal component solutions. These results go beyond the scope of the article. Researchers working with this data should conduct further exploration of the data structure.

## Limitations

None.

## Ethics Statement

The authors declare that the research was carried out in accordance with the Declaration of Helsinki. Informed consent was obtained from all the participants before they filled the online questionnaire. All the data were anonymized.

## CRediT authorship contribution statement

**Jiří Čeněk:** Conceptualization, Methodology, Formal analysis, Data curation, Writing – original draft. **Marek Čejka:** Funding acquisition, Writing – review & editing. **Vladimir Đorđević:** Conceptualization, Writing – review & editing. **Martin Hrabálek:** Conceptualization, Writing – review & editing. **Petra Mlejnková:** Conceptualization, Writing – review & editing. **Ondřej Mocek:** Conceptualization, Writing – review & editing.

## Data Availability

A representative dataset on Czech and Slovak pre/war attitudes to the West and to the Russian Federation (Original data) (OSF)
